# First Report of Coexistence of Three Different MDR Plasmids, and That of Occurrence of IMP-Encoding Plasmid in *Leclercia adecarboxylata*

**DOI:** 10.3389/fmicb.2019.02468

**Published:** 2019-11-05

**Authors:** Zhe Yin, Lingfei Hu, Qiaoxiang Cheng, Xiaoyuan Jiang, Yanan Xu, Wenhui Yang, Huiying Yang, Yuee Zhao, Bo Gao, Jinglin Wang, Erhei Dai, Dongsheng Zhou

**Affiliations:** ^1^State Key Laboratory of Pathogen and Biosecurity, Beijing Institute of Microbiology and Epidemiology, Beijing, China; ^2^Department of Laboratory Medicine, The Fifth Hospital of Shijiazhuang, Hebei Medical University, Shijiazhuang, China

**Keywords:** *Leclercia adecarboxylata*, multidrug resistance, plasmid, mobile elements, *bla*_IMP–8_

## Abstract

Three different MDR plasmids p16005813A, p16005813B, and p16005813C, which carried a total of 18 non-redundant resistance genes or gene loci, were identified in a single clinical isolate of *Leclercia adecarboxylata*. The p16005813A backbone showed very low levels of identity to all DNA sequences available in public databases and carried a *repA* gene that could not assigned into any of known incompatibility groups. The IncFII-family p16005813B and pECAZ161_KPC had essentially identical backbones. p16005813C belonged to an IncR single-replicon plasmid. p16005813A, p16005813B, and p16005813C harbored three different novel MDR regions as their sole accessory modules. The MDR region of p16005813B manifested as Tn*6505*, which was generated from insertion of *bla*_IMP–8_-carrying In655 instead of In4 into the Tn*1696* backbone. Other key antibiotic resistance elements included Tn*2*, IS*26*–*mph(A)*–*mrx*–*mphR(A)*–IS*6100* unit, *chrA* region, In27, and *aacC2*–*tmrB* region in the MDR region of p16005813A, and ΔTn*9* carrying *catA1*, In609, and IS*26*–*tetA*(C)–*tetR*(C)–IS*26* unit in the MDR region of p16005813C. This was the first report of coexistence of three different MDR plasmids, and that of occurrence of IMP-encoding plasmid and *bla*_IMP–8_ gene in *L. adecarboxylata*.

## Introduction

*Leclercia adecarboxylata*, a Gram-negative rod of the *Enterobacteriaceae* family, exists widely in nature and shares many biochemical features with *Escherichia coli* (Anuradha, 2014; Spiegelhauer et al., 2018). As an extremely rare human pathogen, *L. adecarboxylata* causes monomicrobial infection in immune suppressed patients and ones with underlying medical conditions, and it is also found as part of a causative agent of polymicrobial infections in immunocompetent subjects, requiring other coinfecting bacteria to establish infection (Anuradha, 2014; Spiegelhauer et al., 2018).

*L. adecarboxylata* is generally susceptible to cephalosporins, carbapenems, tetracyclines, aminoglycosides, quinolones, and chloramphenicol (Stock et al., 2004). Nevertheless, *L. adecarboxylata* isolates have evolved to acquire foreign antibiotic resistance genes, which encode extended-spectrum β-lactamases (ESBLs) SHV-12 (Mazzariol et al., 2003) and CTX-M-3 (Shin et al., 2012), and carbapenemases IMP-1 (GenBank accession number KJ531212), IMP-4 (Betteridge et al., 2013; Leung et al., 2013), KPC-2 (Geffen et al., 2013; Weingarten et al., 2018), NDM-1 (Sun et al., 2015; Hoyos-Mallecot et al., 2017; Riazzo et al., 2017) and VIM-1 (Papagiannitsis et al., 2013; Papousek et al., 2017); from these strains, one NDM-encoding plasmid pP10164-NDM (accession number KP900016) (Sun et al., 2015), one VIM-encoding pLec-476cz (accession number KY320277) (Papousek et al., 2017), and several KPC-encoding plasmids (Weingarten et al., 2018) have been fully sequenced.

Only a small amount of MDR *L. adecarboxylata* strains, each harboring multiple acquired resistance genes, have been reported (Shin et al., 2012; Garcia-Fulgueiras et al., 2014; Sun et al., 2016). Notably, complete genome sequencing has been applied to only one of those MDR strains designated P10164, which harbors pP10164-NDM that is not a MDR plasmid (Sun et al., 2015), and two MDR plasmids pP10164-2 and pP10164-3 (accession numbers KX710093 and KX710094, respectively), as shown in our previous study (Sun et al., 2016). This follow-up study disclosed co-occurrence three different multi-drug resistant (MDR) plasmids, p16005813A (unknown incompatibility group), p16005813B (IncFII), and p16005813C (IncR), containing a total of 18 non-redundant resistance genes or gene loci, in a single clinical *L. adecarboxylata* isolate.

## Materials and Methods

### Bacterial Isolates

*L. adecarboxylata* 16005813 was isolated in 2016 from a sputum specimen of an infant with pneumonia in a public hospital in Ningbo, China.

### Genomic DNA Sequencing and Annotation

Genomic DNA was isolated from the 16005813 isolate using an DNeasy UltraClean Microbial Kit or a Large Construct Kit (Qiagen, NW, Germany), and then sequenced from a sheared DNA library with average size of 15 kb (ranged from 10 to 20 kb) on a PacBio RSII sequencer (Pacific Biosciences, CA, United States), as well as a paired-end library with an average insert size of 400 bp (ranged from 150 to 600 kb) on a HiSeq sequencer (Illumina, CA, United States). The paired-end short Illumina reads were used to correct the long PacBio reads utilizing *proovread* (Hackl et al., 2014), and then the corrected PacBio reads were assembled *de novo* utilizing *SMARTdenovo*^1^. Open reading frames (ORFs) and pseudogenes were predicted using *RAST* 2.0 (Brettin et al., 2015) with default parameters, combined with *BLASTP/BLASTN* (Boratyn et al., 2013) searches against the *UniProtKB/Swiss-Prot* database (Boutet et al., 2016) and the *RefSeq* database (O’leary et al., 2016). Annotation of resistance genes, mobile elements, and other features was carried out using the online databases including *CARD* (Jia et al., 2017), *ResFinder* (Zankari et al., 2012), *ISfinder* (Siguier et al., 2006), *INTEGRALL* (Moura et al., 2009), and *Tn Number Registry* (Roberts et al., 2008). Multiple and pairwise sequence comparisons were performed using *MUSCLE* 3.8.31 (Edgar, 2004) and *BLASTN*, respectively. Gene organization diagrams were drawn in *Inkscape* 0.48.1^2^.

### Plasmid Transfer

Plasmid conjugal transfer experiments were carried out with the rifampin-resistant *E. coli* EC600 being used as a recipient and the 16005813 isolate as a donor, as described previously (Jiang et al., 2017). Bacteria were spotted on Muller-Hinton agar (BD Biosciences, NJ, United States) plates containing 1000 μg/ml rifampin together with 10 μg/ml azithromycin, 2 μg/ml meropenem or 20 μg/ml tetracycline, for selecting an *E. coli* transconjugant that carried *mph* (A) (p16005813A), *bla*_IMP_ (B) (p16005813B), or *tet* (C) (p16005813C), respectively. Plasmid electroporation experiments were carried out as described previously (Ouyang et al., 2018). Bacteria were spotted on Super Optimal Broth agar plates containing 10 μg/ml azithromycin, 2 μg/ml meropenem or 20 μg/ml tetracycline, for selecting an *E. coli* electroporant that carried p16005813A, p16005813B, or p16005813C, respectively. Each transconjugant or electroporant was further confirmed by PCR detection of *rep* (replication initiation) and/or *bla*_IMP–8_ genes carried by relevant plasmids, followed by sequencing of PCR amplicons using the primers for PCR amplification (Supplementary Table S1).

### Biochemical Assays and Antimicrobial Susceptibility Testing

Activity of carbapenemases in bacterial cell extracts was determined by a modified Carba NP test (Feng et al., 2016). Bacterial antimicrobial susceptibility was tested by bioMérieux VITEK 2 and interpreted according to the Clinical and Laboratory Standards Institute (CLSI) guidelines.

### Nucleotide Sequence Accession Numbers

The complete sequence of plasmids p16005813A, p16005813B, and p16005813C, and that of the 16005813 chromosome, were submitted to GenBank under accession numbers MK036891, MK036884, MK036885, and CP036199, respectively.

## Results and Discussion

### Overview of Sequenced Plasmids

**FIGURE 1:**
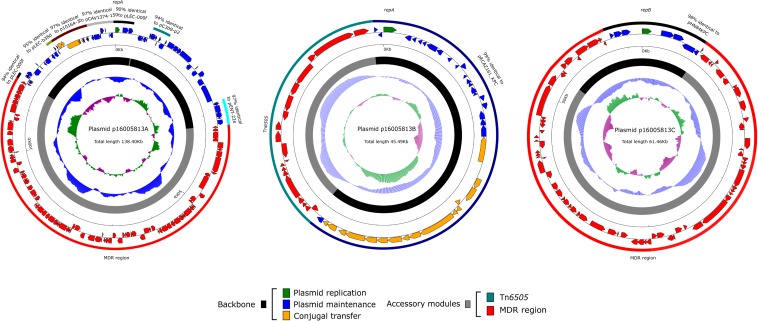
Plasmid schematic maps. Genes are denoted by arrows, and the backbone and accessory module regions are highlighted in black and color, respectively. The innermost circle presents GC-skew [(G–C)/(G + C)], with a window size of 500 bp and a step size of 20 bp. The next-to-innermost circle presents GC content.

The 16005813 isolate carried three plasmids p16005813A, p16005813B, and p16005813C, which exhibited circularly closed sequences 138,399 bp, 45,490 bp, and 61,463 bp in length and contained 149, 56, and 76 ORFs, respectively (Table 1). Each plasmid contained the backbone (responsible for plasmid replication and maintenance, and/or conjugal transfer), with insertion of a single accessory module (acquired DNA region associated and bordered with mobile elements) (Table 1 and Figure 1). A total of 18 non-redundant genes or gene loci, which were involved in resistance to β-lactams including carbapenems (*bla*_IMP–8_, *bla*_CTX–M–9_ and *bla*_TEM–1_), aminoglycosides (*aacC2, aacA4*, and *aadA2*), tetracyclines [*tet* (C)], chloramphenicol (*catA1* and *catB8*), macrolides [*mph* (A)], trimethoprim (*dfrA12*), sulfonamide (*sul1*), quaternary ammonium compounds (*qacED1*), chromate (*chrA*), tunicamycin (*tmrB*), copper (*cop*), mercury (*mer*) and arsenic (Zankari et al., 2012), were found in these three plasmids (Table 2). The 16005813 chromosome carried several putative intrinsic resistance genes (including a fosfomycin resistance gene, and several gene loci encoding putative MDR proteins), but harbored none of accessory resistance regions (Supplementary Table S2).

**TABLE 1:** Major features of plasmids analyzed.

**Category**	**Plasmids**		
	**p16005813A**	**p16005813B**	**p16005813C**
Incomparability group	Unknown	IncFII	IncR
Total length (bp)	138,399	45,490	61,463
Total number of ORFs	149	56	76
Mean G + C content,%	52.5	49.3	53.2
Length of the backbone (bp)	55,270	28,546	9,191
Accessory module	MDR region	Tn*6505*	MDR region

**TABLE 2:** Drug resistance genes in plasmids analyzed.

**Plasmid**	**Resistance gene**	**Resistance phenotype**	**Nucleotide position**	**Region located**
p16005813A	*bla*_TEM–1_	β-lactam resistance	47687.48547 75823.76683 103910.104770	
	*mer* locus	Mercuric resistance	50156.54118 78292.82254	
	*mph(A)*	Macrolide resistance	57038.57943 85174.86079	
	*sul1*	Sulfonamide resistance	64581.65420 92668.93507	
	*qacED1*	Quaternary ammonium compound resistance	65414.65761 93501.93848	MDR region
	*aadA2*	Aminoglycoside resistance	65925.66704 94012.94791	
	*chrA*	Chromate resistance	61562.62767 89698.90902	
	*dfrA12*	Trimethoprim resistance	67124.67621 95211.95708	
	*aacC2*	Aminoglycoside resistance	69991.70851 98078.98938	
	*tmrB*	Tunicamycin resistance	70864.71406 98951.99493	
	*sil* locus	Silver resistance	33066.43739 104918.106694	Plasmid backbone
	*cop* locus	Copper resistance	107992.115527	
	*ars* locus	Arsenic resistance	133081.135957	
p16005813B	*mer* locus	Mercuric resistance	27907.31883	Tn*6505*
	*bla*_IMP–8_	β-lactam resistance	33644.34384	
	*aacA4*	Aminoglycoside resistance	34483.35037	
p16005813C	*mer* locus	Mercuric resistance	16802.20808	MDR region
	*catA1*	Chloramphenicol resistance	26717.27376	
	*bla*_CTX–M–9_	β-lactam resistance	33236.34087	
	*sul1*	Sulfonamide resistance	36384.37223	
	*catB8*	Chloramphenicol resistance	37721.38353	
	*aacA4*	Aminoglycoside resistance	38765.39342	
	*tet*(C)	Tetracycline resistance	42678.43868	
				

p16005813A, p16005813B, and p16005813C could be transferred from the 16005813 isolate into TOP10 through electroporation, generating the electroporations p16005813A-TOP10, p16005813B-TOP10, and p16005813C-TOP10 (Table 3), respectively. Successful conjugation transfer of p16005813B, but not p16005813A and p16005813C, into EC600 generated the transconjugant p16005813B-EC600, which was consistent with the fact that only p16005813B carried a complete set of conjugal transfer genes. Strains 16005813, p16005813B-TOP10, and p16005813B-EC600 had metallo-β-lactamase activity and were resistant to ceftazidime and imipenem (Table 3), which was resulted from encoding of IMP enzyme by p16005813B.

**TABLE 3:** Antimicrobial drug susceptibility profiles.

**Antibiotics**	**Minimum inhibitory concentration (mg/L)/antimicrobial susceptibility**						
	**16005813**	**p16005813A-TOP10**	**p16005813B-EC600**	**p16005813B-TOP10**	**p16005813C-TOP10**	**TOP10**	**EC600**
Cefazolin	≥64R	8R	≥64R	≥64R	≥64R	≤4S	≤4S
Cefuroxime	≥64R	4S	≥64R	≥64R	≥64R	≤4S	16I
Ceftriaxone	≥64R	≤1S	≥64R	≥64R	≥64R	≤1S	≤1S
Ceftazidime	≥64R	≤1S	≥64R	≥64R	16R	≤1S	≤1S
Imipenem	8R	≤1S	≥16R	≥16R	≤1S	≤1S	≤0.25S
Levofloxacin	1S	≤0.25S	1I	≤0.5S	≤0.25S	0.5S	≤16S
Gentamicin	≥16R	≥16R	≥16R	≥16R	≥16R	≤1S	≤1S
Trimethoprim/sulfamethoxazole	≥320R	≥320R	≥320R	≥320R	≤20S	≤0.25S	≤20S
Tetracycline	≥16R	≤1S	≤1S	≤1S	≥16R	≤1S	≤1S

*S, sensitive; R, resistant; I, intermediate.*

### Plasmid Backbone Regions

The p16005813A backbone showed very low levels of identity to all DNA sequences available in public databases. The replication initiation gene *repA* of p16005813A showed 100% BLAST coverage and 94% nucleotide identity to the counterparts in pLEC-5e18 (accession number CP026390) and pLEC-000f (accession number CP026170) from *L. adecarboxylata*; all these RepA proteins could not assigned into any of known incompatibility groups. A 121-bp region containing six imperfect direct repeats of GTGtGTcataacATG was located 89-bp upstream of *repA* of p16005813A, and might function as RepA-binding iterons. Key determinants for plasmid maintenance include a type Ia partitioning system ParABC, two distinct type II toxin-antitoxin systems VapBC and ParDE involved in post-segregational killing. Residual conjugal transfer determinants TrbA, MobC and mutated NikAB were found, which was consistent with the p16005813A’s nature of not self-transmissible.

p16005813B and pECAZ161_KPC (accession number CP019010) had essentially identical backbones and each of them carried a single accessory module, but the two accessory modules were different with respective to resistance genes and mobile elements harbored, and inserted at two different sites of the backbone. Key backbone genes of p16005813B include a novel replication initiation gene *repA* belonging to the IncFII family, *ccdBA* encoding a type II toxin-antitoxin system, and a set of P-type type IV secretion system genes (*tviF3/12/13/15/17*) involved in conjugal transfer.

The p16005813C backbone was composed of *repB* (replication initiation) and its iterons, *parAB* (type Ia partitioning), *umuCD* (SOS mutagenesis), and *resD* (multimer resolvase), but lacked *retA* (group IIB intron-encoding reverse transcriptase), and *vagCD* (toxin-antitoxin) compared to the IncR single-replicon reference plasmid pHN84KPC (accession number KY296104) with the most complete IncR backbone. IncR single-replicon plasmids including p16005813 lacked conjugal transfer genes and thus were not self-transmissible (Chen et al., 2014; Compain et al., 2014).

### The MDR Regions

**FIGURE 2:**
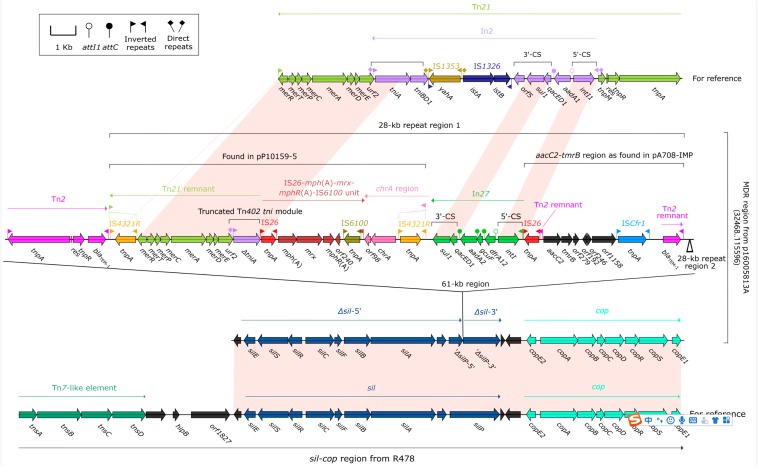
Organization of the MDR region of p16005813A and comparison with related regions. Genes are denoted by arrows. Genes, mobile elements and other features are colored based on function classification. Shading denotes regions of homology (>95% nucleotide identity). Numbers in brackets indicate e nucleotide positions within corresponding plasmids. The accession numbers of the *sil–cop* region from R478, Tn*21*, and Tn*2* for reference are BX664015, AF071413, and HM749967, respectively.

Each of p16005813A, p16005813B and p16005813C harbored a MDR region as the sole accessory module. The MDR region (83.1 kb in length, Figure 2) of p16005813A was generated from insertion of a 61-kb region into a truncated (losing of *tnsABCD*) version of the *sil–cop* region, which was originally found in the IncHI2 reference plasmid R478 (accession number BX664015) (Gilmour et al., 2004) and composed of a Tn*7*-like core transposition module *tnsABCD* together with the silver (*sil*) and copper (*cop*) resistance loci. Various derivatives of the *sil–cop* region were presented in IncHI2 plasmids (Sun et al., 2016; Liang et al., 2017). The 61-kb region comprised Tn*2*, and two 28-kb repeat regions, each of which harbored a *mer*-containing Tn*21* remnant + IS*26–mph*(A)–*mrx–mphR*(A)–IS*6100* unit + *chrA* region as found in pP10159-5 (Ouyang et al., 2018), In27, and *aacC2–tmrB* region as found in pA708-IMP (accession number MF344567).

**FIGURE 3:**
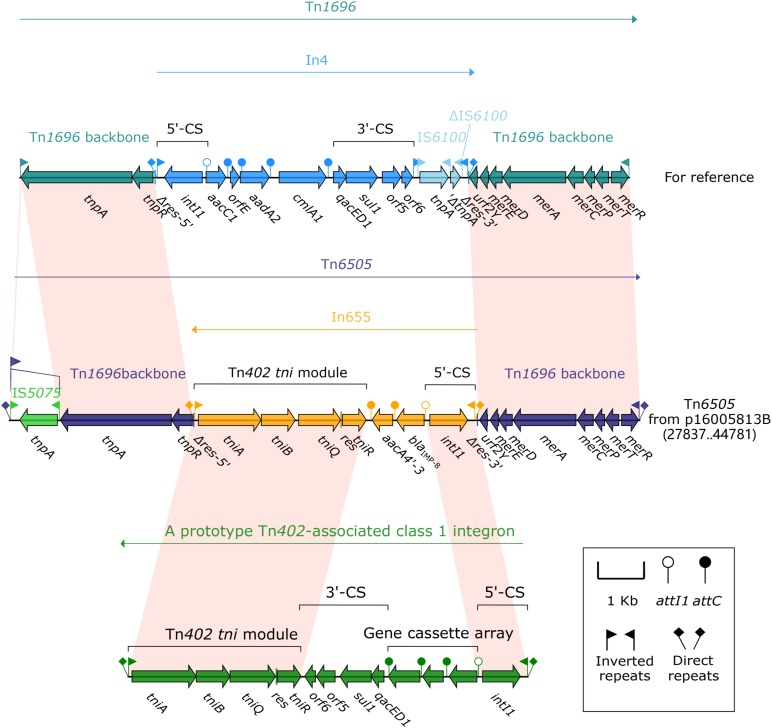
Organization of the MDR region (Tn*6505*) of p16005813B and comparison with related regions. Genes are denoted by arrows. Genes, mobile elements and other features are colored based on function classification. Shading denotes regions of homology (>95% nucleotide identity). Numbers in brackets indicate e nucleotide positions within corresponding plasmids. The accession number of Tn*1696* for reference is U12338.

Tn*6505* (16.9 kb in length, Figure 3) from p16005813B was a novel derivative of Tn*1696* (Partridge et al., 2001) belonged to the Tn*21* subgroup of Tn*3* transposon family, and flanked by 5 bp direct repeats (DRs; target site duplication signals for transposition). Tn*6505* differed from Tn*1696* by (i) insertion of *bla*_IMP–8_-carrying In655 instead of In4 at the same position within the resolution (*res*) site, and (ii) interruption of IRL_Tn*6505*_ (inverted repeat right of Tn*6505*) by IS*5075* that was a hunter of terminal IRs of Tn*21* subgroup transposons (Partridge and Hall, 2003).

**FIGURE 4:**
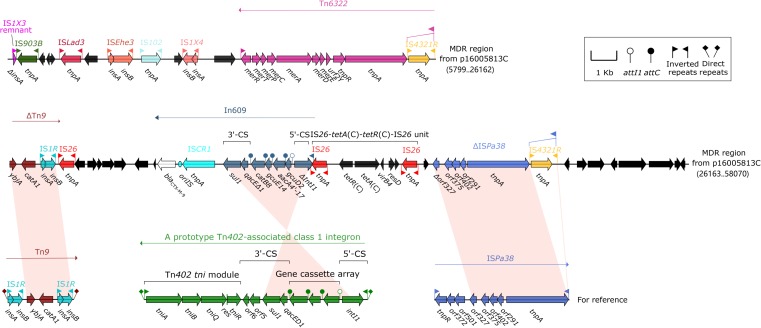
Organization of the MDR region of p16005813C and comparison with related regions. Genes are denoted by arrows. Genes, mobile elements and other features are colored based on function classification. Shading denotes regions of homology (>95% nucleotide identity). Numbers in brackets indicate e nucleotide positions within corresponding plasmids. The accession numbers of Tn*9* and IS*Pa38* for reference are LN830952, and MH909331, respectively.

The MDR region (52.2 kb in length, Figure 4) from p16005813C carried at least four resistance modules, namely Tn*6322* (Sun et al., 2016), ΔTn*9* carrying *catA1* (Alton and Vapnek, 1979), In609, IS*26–tetA*(C)–*tetR*(C)–IS*26* unit (Sun et al., 2016). Tn6*322* was composed of a Tn*21* core transposition module *tnpAR–res* together with a *mer* locus, and its IRL was interrupted by IS*4321R* (a homolog of IS*5075*). In609 was a complex class 1 integron carried variable region 1 (VR1: gene cassette *gcuD2*–*aacA4′-17*–*gcuE14*–*catB8*) and VR2 (*bla*_CTX–M–9_).

## Conclusion

This is the first report of coexistence of three different MDR plasmids, and that of occurrence of IMP-encoding plasmid and *bla*_IMP–8_ gene in *L. adecarboxylata*. *bla*_IMP–8_ was located in In655, representing an ancestral Tn*402*-associated integron (containing a complete *tni*_*Tn*_*_402_* core transposition module) at stage I of evolution as defined previously (Jiang et al., 2017). The three co-existent MDR plasmids carried a large amount of resistance genes, making the relevant *L. adecarboxylata* isolate tend to be extensively drug resistant. Epidemiological investigation of multi-drug resistant *L. adecarboxylata* needed to be carried out in China.

## Data Availability Statement

The datasets generated for this study can be found in the complete sequence of plasmids p16005813A, p16005813B, and p16005813C, and that of the 16005813 chromosome, were submitted to GenBank under accession numbers MK036891, MK036884, MK036885, and CP036199, respectively.

## Ethics Statement

This study uses the clinical bacterial isolate obtained from a public hospital in Ningbo, China. The study needs not to be reviewed or approved by the ethics committee of the hospital, because the bacterial isolate involved in this study was part of the routine hospital laboratory procedure. The research involving biohazards and all related procedures were approved by the Biosafety Committee of the Beijing Institute of Microbiology and Epidemiology.

## Author Contributions

DZ and ED conceived the study and designed experimental procedures. ZY, LH, QC, XJ, and YX performed the experiments. ZY, LH, DZ, and ED analyzed the data. WY, HY, YZ, BG, and JW contributed to reagents and materials. DZ, ZY, LH, and ED wrote the manuscript.

## Conflict of Interest

The authors declare that the research was conducted in the absence of any commercial or financial relationships that could be construed as a potential conflict of interest.

## Footnotes

1 https://github.com/ruanjue/smartdenovo

2 https://inkscape.org/en/
