# Supplementary material for: First Report of Coexistence of Three Different MDR Plasmids, and That of Occurrence of IMP-Encoding Plasmid in Leclercia adecarboxylata
Source: Front Microbiol. 2019 Nov 5;10:2468. doi: 10.3389/fmicb.2019.02468 (PMC6848029; doi:10.3389/fmicb.2019.02468)
Supplement: Supplementary file 1 [file Table_1.pdf]

Table S1 | PCR detection of *bla*$_{\mathbf{IMP\text{-}8}}$ and *rep* genes

Target gene	Primers	S-A	A-T
bla$_{IMP\text{-}8}$	IMP-F: 5'-CAGCAACGATGTTACGCAGC-3'<br>IMP-R: 5'-AGCAGCCGTAGAGCTTTTGG-3'	1541	52
repA$_{p16005813A}$	16005813A-F: 5'-ACCTCGAATGGATTCTCAACACA-3'<br>16005813A-R: 5'-CGGCATGAAGGCTAAAGCTC-3'	997	57
repA$_{p16005813B}$	16005813B-F: 5'-CAAATCAACCCCTGTTCGCC-3'<br>16005813B-R: 5'-GCAAAGCCTCCGTTGATACG-3'	958	56
repB$_{p16005813C}$	16005813C-F: 5'-TGGGATACGCATCAACAAGC-3'<br>16005813C-R: 5'-ACAGGTCAAGTTAGGCAAGCA-3'	986	52

S-A: Size of amplicon (bp); A-T: Annealing temperature (℃).
