# Supplementary material for: First Report of Coexistence of Three Different MDR Plasmids, and That of Occurrence of IMP-Encoding Plasmid in Leclercia adecarboxylata
Source: Front Microbiol. 2019 Nov 5;10:2468. doi: 10.3389/fmicb.2019.02468 (PMC6848029; doi:10.3389/fmicb.2019.02468)
Supplement: Supplementary file 2 [file Table_2.pdf]

TABLE S2 | Putative resistance genes in the 16005813 chromosome

Gene	Gene product	Nucleotide position
**Fosfomycin resistance**		
fosA	Fosfomycin resistance protein	98056..98481
**Multidrug resistance**		
sugE	Multidrug efflux system protein	252189..252506
emrD	Multidrug resistance protein	583942..585126
mdtL	Multidrug efflux system protein	614741..615916
emrR	Transcriptional repressor	1641550..1642080
bcr	Multidrug efflux system	2203626..2204822
mdtQ	Multidrug resistance outer membrane protein	2266027..2267445
mdtH	Multidrug resistance protein	3727403..3728611
mdtG	Drug efflux system protein	3736448..3737731

Genes were annotated by using CDD (NCBI Conserved Domains Database).
